# Risk factors of malnutrition among preschool children in Terengganu, Malaysia: a case control study

**DOI:** 10.1186/1471-2458-14-785

**Published:** 2014-08-03

**Authors:** Hui Jie Wong, Foong Ming Moy, Sulochana Nair

**Affiliations:** Department of Development Studies, Faculty of Economics and Administration, University of Malaya, Kuala Lumpur, 50603 Malaysia; Julius Centre University of Malaya, Department of Social and Preventive Medicine, Faculty of Medicine, University of Malaya, Kuala Lumpur, 50603 Malaysia; Binary University of Management & Entrepreneurship, No 1, IOI Business Park, Persiaran Puchong Jaya Selatan, Bandar Puchong Jaya, Selangor, 47100 Malaysia

**Keywords:** Children, Malnutrition, Risk factor, Case control, Malaysia

## Abstract

**Background:**

Childhood malnutrition is a multi-dimensional problem. An increase in household income is not sufficient to reduce childhood malnutrition if children are deprived of food security, education, access to water, sanitation and health services. The aim of this study is to identify the characteristics of malnourished children below five years of age and to ascertain the risk factors of childhood malnutrition in a state in Malaysia.

**Methods:**

A case control study was conducted in the maternal and child health clinics in five districts of Terengganu, Malaysia from April to August 2012. Case was a child with moderate to severe malnutrition with z-scores < −2SD from the median of WHO reference. Control was a child without malnutrition with z-scores between -2SD and +2SD and the age matched with case’s. Face to face interviews with the respective child’s mother and measurements of the respective child’s weight and height were carried out. Information on socio-economic characteristics, household food security status, child’s dietary intake, caregivers’ practices and resources were enquired. Univariate and multivariate logistic regression analyses were conducted. Crude odds ratio and adjusted odds ratio with 95% confidence interval were calculated.

**Results:**

A total of 274 children with 137 cases and 137 controls were recruited. All respondents were Malays. Among the cases, a larger proportion of them was female and originated from low income families. After adjusting all confounders, childhood malnutrition was significantly associated with number of children (aOR: 5.86, 95% CI: 1.96, 17.55), child hunger (aOR: 16.38, 95% CI: 1.34,199.72), dietary energy intake (aOR: 0.99, 95% CI: 0.98, 0.99), protein intake (aOR: 1.06, 95% CI: 1.01, 1.12), vitamin A intake (aOR: 0.999, 95% CI: 0.997, 1.00), low birth weight (aOR: 6.83, 95% CI: 1.62, 28.89), frequent illness (aOR: 2.79, 95% CI: 1.06, 7.31), and history of worm infection (aOR: 3.48, 95% CI: 1.25, 9.70).

**Conclusions:**

Lower socio-economic status, household food insecurity, and poor child caring practices were associated with childhood malnutrition. Besides implementation of programmes focusing on poverty reduction, community based nutrition and hygiene education with extensive family planning and de-worming programmes should be intensified to improve both mother and children’s nutritional status.

## Background

Globally, it was estimated that one in every three preschool children is malnourished [1]. In 2011, an estimation of 165 million children under-five years of age were underweight, 101 million were stunted and 52 million were wasted [1]. Childhood malnutrition is influenced by multidimensional factors. These factors vary from biological, behavioural and environmental [2–4]. A number of studies demonstrated that childhood malnutrition is strongly rooted in poverty [5–8]. However, the relationship between poverty and childhood malnutrition is rather complex. High household income may not guarantee a satisfactory nutritional outcome of the children if households are lacking of care, dietary quality and health care access [9–11]. Malnutrition affects both poor and non-poor households [12, 13]. Malnutrition will persist despite rapid income growth if more effective approach to combat the problem is absent.

Malaysia is an upper middle income country with good improvement in economy and reduction of poverty. However, problems of childhood malnutrition still persist especially in the rural communities [12–15]. While the problems and prevalence of childhood malnutrition in poor communities of Malaysia are well-documented, its specific determinants are not well understood. Previous local studies focused mainly on the socio-economic status differences measured in terms of income, wealth status and housing index [12, 13]. Very few studies explored the impact of other determinants such as dietary intake [14, 15], food security, caring practices [14] and role of women on children’s nutritional status. Therefore, it is the aim of this study to identify the characteristics of malnourished children and to ascertain the risk factors which contribute to childhood malnutrition in a state in Malaysia.

## Methods

### Study setting and design

A case control study was carried out in the state of Terengganu from April to August 2012. This state was chosen as it has the highest under-five mortality rate in Malaysia in 2011 [16]. It was also one of the top five states in Peninsular Malaysia receiving food basket assistance for malnourished children between 2009 to 2011 [17]. Terengganu is situated in north-eastern Peninsular Malaysia and is bordered by Kelantan, Pahang and the South China Sea. Terengganu is divided into seven administrative districts which include Kemaman, Dungun, Marang, Hulu Terengganu, Kuala Terengganu (capital), Setiu, and Besut. As of 2010, Terengganu has a population of 1,035,977 [18]. Malays formed the largest ethnic group with 95.1%, followed by Chinese (2.6%), Indians (0.2%) and other ethnic groups (2.1%) [18].

This study was conducted in the maternal and child health (MCH) clinics. Out of the seven districts in Terengganu, five districts with the highest number of malnourished children in 2011 were selected. The selected districts were Dungun, Marang, Kuala Terengganu, Hulu Terengganu and Besut. There are thirty nine health clinics in the selected five districts. Based on the number of children visiting health clinics in 2011, one health clinic with the highest number of children attendance was selected from each of the district.

### Recruitment of respondents

All children less than five years old who visited the MCH clinics during the data collection period were screened using their anthropometric data. Malnourished children were first identified and then selected as cases based on the inclusion and exclusion criteria. Cases were children with a diagnosis of moderate to severe malnutrition regardless of the types of malnutrition (can be either underweight, stunting, wasting or a combination of all) with z-scores < −2SD from the median of WHO reference [19]. Controls were children without malnutrition (have normal anthropometric readings of weight for age, height for age, and weight for height but were not overweight or obese) with z-scores between -2SD and +2SD (−2SD ≤ z-scores ≤ +2SD) [19]. Controls were age-matched with cases and were selected from the same health clinic on first come first serve basis. Children with mental retardation, physical challenges, serious illnesses or born as preterm babies (less than 37 weeks of gestation) were excluded from the study. After measurements, mothers of the respondents were interviewed by the principal investigator based on a validated questionnaire. Only children accompanied by their own mothers were recruited to avoid recall bias. One child corresponded to one household unit.

### Sample size calculation

Study by Chee et al. [12] demonstrated the probability of exposure to poverty among controls was 0.39 [12] and the odds ratio for poor household with malnourished children as compared to non-poor household was 2.15 [12]. Correlation coefficient for exposure between matched cases and controls was set at 0.2 [13]. Using the sample size calculator of Open Source Epidemiologic Statistics for Public Health (version 3.01), with 80% power, significant level at 0.05, 220 respondents were required. Considering 24% of non-response rate, an estimated 137 cases with 137 controls were needed giving a total number of 274 respondents.

### Study variables

#### Dependent variables

The dependent variables of this study were gender-specific anthropometric z-scores of weight-for-height, weight-for-age and height-for-age. Based on the z-scores, a child was classified into two categories: i) undernourished (case); or ii) well-nourished (control).

### Independent variables

This study identified three domains affecting children’s nutritional status. Each domain comprised of several variables as presented below:

- *Household socio-economic characteristics:* parental age, parental ethnicity, parental working status, parental education level, paternal working industry, total household members, sex of children, total number of children, household poverty status, type of toilet and sources of drinking water
- *Household food security status and child’s dietary intake:* household food security status and dietary nutrients intake such as energy, protein, vitamin A and iron
- *Caregivers’ practices and resources:* duration of exclusive breast feeding, age of weaning, child’s birth weight, frequency of child’s illness (flu/diarrhoea/fever), history of child’s worm infections, maternal use of family planning, mother’s autonomy in decision making, maternal ownership of asset and body mass index (BMI) of mother

### Questionnaires

Data on socio-demographic characteristics, household access to facilities, household poverty status, children’s health status and children’s dietary intake were collected using the questionnaire developed and validated by Cheah et al. [20]. The questionnaires were administered by the principal investigator through face to face interview with the child’s mother. Most of the variables under socio-demographic dimension were categorized into two or more categorical variables as the following:

i. Ethnicity: Malay, Chinese, Indian or others
ii. Paternal working status: jobless or retired, government sector, private sector, or self-employed
iii. Maternal working status: housewife or working
iv. Paternal working industry: agriculture, mining, manufacturing, construction, or services
v. Parental education level: primary education and less, secondary education, or tertiary education
vi. Total household members: ≤5 persons or > 5 persons
vii. Sex of children: female or male
viii. Total number of children: 3 children and below or 4 children and above
ix. Type of toilet: flush toilet or pour/pit toilet
x. Sources of drinking water: pipe water inside house (yes or no)

In addition, parental age was reported as continuous variable and paternal occupation was recorded. Household poverty status was determined by household income. The household income per capita was compared with national poverty line income (PLI) [21]. Household was categorized into three different groups: i) poor (income per capita ≤ RM198); ii) low income (RM198 < income per capita ≤ RM357); and iii) non-poor (income per capita > RM357).

Household food security was assessed using Radimer/Cornell Hunger and Food Insecurity instrument which has been validated and shown to be applicable in Malaysia [22]. Using the ten questions in the instrument, households were assigned to mutually exclusive groups representing increasing severity of food insecurity: i) household food secure; ii) household food insecure; iii) individual food insecure; and iv) child hunger.

Dietary intakes of children were obtained from an interactive 24-hours dietary recall. Mothers of the children were asked to recall all food and beverages consumed by their children over the past 24-hours. Respondents were probed for the types of food, food preparation methods, ingredients and portion sizes. Face to face interview was conducted using household measures to facilitate quantification of portion sizes consumed. For food which is not available in the database of Nutritionist Pro™ Nutrition Analysis Software, information on energy and nutrients content on its packaging was entered into the software for analysis. Similar nutrients content were matched to existing food in the database. In order to ensure the quality of food intake data, the number of under reporters were checked by using the Goldberg cut off technique [23]. First, the basal metabolic rate (BMR) of each child was calculated by using the Schofield Equation. Then, the ratio of energy intake (EI) to basal metabolic rate (EI:BMR) was calculated. The individual and mean of EI:BMR was evaluated for both cases and controls based on a cut-off value for low-energy reporters from the previous published study. With reference to a study conducted in Dortmund, Germany on children aged between one to five years old, the cut-off value for low-energy reporters was set at less than 0.97 [24].

Besides, information on caregivers’ practices and resources was collected. Using the cut off points of BMI for Asian population, the caregivers were grouped into three categories of BMI: underweight (<18.5 kg/m^2^), normal (18.5-22.9 kg/m^2^), overweight and obese (≥23 kg/m^2^). Mothers of the children were asked if they had autonomy in determining household expenditure in the family and whether they were involved in decision making. They were also asked whether they possessed any personal asset. The ownership of asset status was categorized into “yes” or “no”.

### Anthropometric measurements

Length and weight of infants or children less than two years old were measured in recumbent position with an electronic paediatric scale (with maximum capacity weight of 12 kg and accuracy of 0.05 kg) and measuring board (with accuracy of 0.1 cm). The measuring scale has a stationary headboard and moveable footboard that is perpendicular to the backboard. Weight and height of the children who were more than two years old and their mothers were measured on a digital scale (with maximum capacity weight of 200 kg and accuracy of 0.1 kg) with an attached wall-mounted stadiometer (with accuracy of 0.1 cm). The digital scale included a side-mounted measuring rod which allows simultaneous measuring and weighing. During measurement, participants were barefoot and wore light clothing. Their bodies were stretched upward to the full extent in the Frankfurt position. Two readings were obtained for each measurement. The mean of two anthropometric measurements was calculated and used for the analysis.

### Ethical considerations

Permission to carry out the study was obtained from the state and district health authorities. The study protocol was reviewed and approved by the Malaysia National Institute of Health and Medical Research Ethics Committee in the Ministry of Health (NMRR-12-477-11611). Informed consent was obtained from the mothers before their children were recruited into the study.

### Statistical analyses

Statistical analyses were performed using the IBM Statistical Program for Social Sciences (SPSS) version 21. The z-score values of weight for age (WAZ), height for age (HAZ) and weight for height (WHZ) were computed using the WHO Anthro (version 3.2.2, 2011) [25]. Nutritionist Pro™ Nutrition Analysis Software was used to analyse energy and nutrients intake. Descriptive analyses were computed to compare the socio-economic characteristics, household food security status, child’s dietary intake, and caregiver variables between cases and controls. Mean and standard deviation were reported for normally distributed continuous variables. Counts and percentages for categorical variables were reported. Chi squared test and *t*-test were used to examine the relationship between childhood malnutrition and study variables. Univariate and multivariate logistic regression analyses were conducted. Crude odds ratios (OR) and adjusted odds ratios (aOR) with 95% confidence intervals (CI) were calculated. P values less than 0.05 were considered statistically significant.

## Results

### Characteristics of the study population

A total of 274 respondents (137 cases age-matched with 137 controls) were recruited. Their mean age was 27.5 months. Almost one third of the respondents were aged between 12 months to 23 months while one quarter of them were aged between 24 months to 35 months. There were more girls (56%) and all respondents were of Malay ethnicity. All of the respondents’ caregivers were their mothers. The mean weight ± standard deviation for the cases and controls was 9.3 ± 2.1 kg and 12 ± 2.9 kg respectively, while their mean height ± standard deviation was 81.1 ± 10.2 cm and 87 ± 11.2 cm respectively. A majority of the cases (76.6%) were moderately malnourished while 23.4% of them were severely malnourished. Among the cases, 71% of them were underweight, 62% were stunted and 38% were wasted.

### Socio-economic characteristics

Table 1 shows the socio-economic characteristics of the respondents. It was found that the mean age of the mothers and fathers of the cases was thirty one and thirty six years old respectively. A majority of the cases’ parents attained secondary education level. More than half of the mothers of the cases were housewives while their fathers were employed by private agencies and worked in the services industry. The number of family members among the cases varied between three and fifteen, with a mean value of five. A majority of the cases had pour or pit toilets and piped water located inside their houses. A higher proportion of the cases was female, resided in poor and low income households and had four siblings in their families as compared with controls. In addition, the proportion of mothers who completed tertiary education and working was lower among the cases. No significant associations were found between parental age, ethnicity, paternal education, household size, type of toilet and sources of drinking water with childhood malnutrition.

**Table 1:** **Comparison of socio-economic characteristics between cases and controls**

Variable	Case (n = 137)		Control (n = 137)		
	Mean ± sd	n (%)	Mean ± sd	n (%)	p value
**Child’s age (month)**					
0-5	5		4		
6-11	8		8		
12-23	19		19		
24-35	30		29		
36-47	40		41		
48-59	54		54		
**Child’s sex**					0.003
Female		88 (64.2)		65 (47.4)	
Male		49 (35.8)		72 (52.6)	
**Mother’s age (years)**	31 ± 6		30 ± 7		0.300
**Father’s age (years)**	36 ± 7		34 ± 7		0.170
**Mother’s ethnicity**					1.000
Malay		137 (100)		137 (100)	
**Father’s ethnicity**					0.990
Malay		130 (99.2)		132 (99.2)	
**Mother’s educational level**					0.003
Primary and less		15 (11)		11 (8.0)	
Secondary		103 (75.2)		83 (60.6)	
Tertiary		19 (13.8)		43 (31.4)	
**Father’s educational level**					0.090
Primary and less		18 (13.7)		8 (6)	
Secondary		89 (68)		94 (70.7)	
Tertiary		24 (18.3)		31 (23.3)	
**Mother’s working status**					0.049
Housewife		91 (66.4)		75 (54.7)	
Working		46 (33.6)		62 (45.3)	
**Father’s working status**					0.310
Jobless or retired		3 (2.3)		1 (0.8)	
Government		17 (13)		29 (21.8)	
Private		69 (52.7)		62 (46.6)	
Self-employed		42 (32)		41 (30.8)	
**Household poverty status (income per capita)**					<0.001
Poor (≤RM198)		37 (27)		18 (13.1)	
Low income (RM198 < x ≤ RM357)		40 (29.2)		27 (19.7)	
Non-poor (>RM357)		60 (43.8)		92 (67.2)	
**Household size**					0.710
5 persons and below		82 (59.9)		85 (62)	
6 person and above		55 (40.1)		52 (38)	
**Number of children**					0.013
3 persons and below		93 (67.9)		111 (81)	
4 persons and above		44 (32.1)		26 (19)	
**Type of toilet**					
Flush toilet		61(44.5)		73 (53.3)	0.150
Pour/pit		76 (55.5)		64 (46.7)	
**Sources of drinking water**					0.420
Pipe water inside house		123 (89.8)		125 (91.2)	

Note. case = moderate to severe malnourished preschool children with z-scores < −2SD from the median of WHO reference based on visits to health clinics of Terengganu, Malaysia between April- August 2012; control = child without malnutrition with z-scores between -2SD and +2SD, age matched and selected from the same health clinic as case; sd = standard deviation; n = total number; primary education and less = attained less than 6 years of school; secondary education = attained 11 years of education; tertiary education = obtained certificate, diploma, first degree and higher degree qualifications at academic and professional fields; poor = income per capita ≤ RM198; low income = RM198 < income per capita ≤ RM357; non-poor = income per capita > RM357; significance level is p < 0.05.

### Household food security and children’s dietary intake

Table 2 presents the household food security status and dietary intake of children. Food insecurity problem was more common among the cases. More than half (62%) of the cases reported that they experienced some kind of food insecurity; with 19% of them reported at household level, 23% at individual level and 20% at child hunger level. These cases had significantly lower intake of energy, protein, vitamin A and iron as compared to the controls (p < 0.05). In this study, it was found that there were 25 (10.5%) of the respondents with EI: BMR ratio of less than 0.97. The mean value of EI:BMR ratio for the cases and controls was 1.26 and 1.56 respectively. Based on this finding, the number of under reporters in this study was acceptable and the data quality of the dietary intake was good.

**Table 2:** **Comparison of household food security status and dietary intake between cases and controls**

Variables	Case (n = 137)		Control (n = 137)		
	Mean ± sd	n (%)	Mean ± sd	n (%)	p value
**Household food security status**					<0.001
Household food secure		52 (38)		74 (54)	
Household food insecure		26 (19)		40 (29.2)	
Individual food insecure		31 (22.6)		21 (15.3)	
Child hunger		28 (20.4)		2 (1.5)	
**Child’s dietary intake**					
Kilocalories (kcal)	715 ± 206		1035 ± 213		<0.001
% TE/RNI	78		109		
Protein (g)	28 ± 11		39.5 ± 13.8		<0.001
% TP/RNI	169		230		
Vitamin A (RE)	387 ± 278		643 ± 388		<0.001
% Vit A/RNI	94		159		
Iron (mg)	6.9 ± 3.8		10.5 ± 4.9		<0.001
% Fe/RNI	165		257		

Note. case = moderate to severe malnourished preschool children with z-scores < −2SD from the median of WHO reference based on visits to health clinics of Terengganu, Malaysia between April - August 2012; control = child without malnutrition with z-scores between -2SD and +2SD, age-matched and selected from the same health clinic as case; sd = standard deviation; n = total number; RNI = recommended nutrients intake; %TE/RNI = percentage of total dietary energy intake as compared to RNI; %TP/RNI = percentage of total dietary protein intake as compared to RNI; % Vit A/RNI = percentage of total dietary vitamin A intake as compared to RNI; % Fe/RNI = percentage of total dietary iron intake as compared to RNI; significance level is p < 0.05.

### Caregivers’ practices and resources

Table 3 shows the respondents’ caregivers’ practices and resources. There were no significant associations between duration of exclusive breast feeding, weaning age and use of health services with childhood malnutrition. Almost half of the mothers used contraceptive methods of birth controls. The differences between cases and controls with regard to their frequency of illness, worm infection, birth weight, maternal BMI and maternal ownership of asset were statistically significant (p < 0.05). One in every four cases (25%) was low birth weight as compared to only 5.1% among controls. Moreover, more than one third of the cases experienced at least one episode of fever, flu or diarrhoea every month. A higher number of cases’ mothers did not own any personal asset and were underweight compared to mothers of controls. Overweight or obesity among mothers, however, was not found to be associated with childhood malnutrition.

**Table 3:** **Comparison of caregivers’ practices and resources between cases and controls**

Variables	Case (n = 137)	Control (n = 137)	
	n (%)	n (%)	p value
**Duration of exclusive breast feeding**			0.080
< 6 months	73 (54.1)	87 (64.4)	
≥ 6 months	62 (45.9)	48 (35.6)	
**Weaning age**			0.780
0-3 months	13 (9.5)	12 (8.8)	
4-6 months	115 (84)	113 (82.5)	
7 months and above	9 (6.5)	12 (8.7)	
**Child’s birth weight**			<0.001
Low birth weight (<2.5 kg)	35 (25.5)	7 (5.1)	
**Frequency of child’s illness (fever/diarrhoea/flu)**			<0.001
Every month	51 (37.2)	20 (14.6)	
Once in 2 months	20 (14.6)	18 (13.1)	
Rarely (>3 months)	66 (48.2)	99 (72.3)	
**History of child’s worm infection (yes)**	43 (31.4)	23 (16.8)	0.005
**Mother’s use of family planning (yes)**	67 (48.9)	77 (56.2)	0.230
**Person who has autonomy in determining household expenses**			0.460
Mother	61 (44.5)	71 (51.8)	
Father	73 (53.3)	64 (46.7)	
Grandparent	3 (2.2)	2 (1.5)	
**Mother’s personally own asset (yes)**	41 (29.9)	66 (48.2)	0.002
**BMI status of the mother**			0.014
Underweight (<18.5 kg/m^2^)	14 (10.2)	2 (1.5)	
Normal weight (18.5-22.9 kg/m^2^)	53 (38.7)	48 (35)	
Overweight/obesity (≥23 kg/m^2^)	70 (51.1)	87 (63.5)	

Note. case = moderate to severe malnourished preschool children with z-scores < −2SD from the median of WHO reference based on visits to health clinics of Terengganu, Malaysia between April- August 2012; control = child without malnutrition with z-scores between -2SD and +2SD, age-matched and selected from the same health clinic as case; BMI = body mass index; significance level is p < 0.05.

### Results of multivariate analyses

The results of multivariate analyses are presented in Table 4 after adjusting for maternal education level, maternal working status, household poverty status, child’s sex, dietary iron intake, maternal ownership of asset and maternal BMI. The strongest predictor of childhood malnutrition was child hunger. The multivariate analyses indicated that households which experienced food insecurity at the child hunger level were found to be 16 times more likely (aOR: 16.38, 95% CI: 1.34-199.72) to have malnourished children. Similarly, households with four children and above (aOR: 5.86, 95% CI: 1.96, 17.55) or households with low birth weight children (aOR: 6.83, 95% CI: 1.62, 28.89) were at higher odds of having malnourished children as compared to their counterparts. In addition, children who were sick every month (aOR: 2.79, 95% CI: 1.06, 7.31) and had a history of worm infection (aOR: 3.48, 95% CI: 1.25, 9.70) were three times more likely to be malnourished as compared to their counterparts. An increased dietary intake of energy and vitamin A were protective factors for childhood malnutrition.

**Table 4:** **Unadjusted and adjusted odds ratios of risk factors for childhood malnutrition: a case control study (n = 274)**

Variables	Crude odds ratio (95% CI)	p value	Adjusted odds ratio ^1^ (95% CI)	p value
**Mother’s educational level**				
Primary and less	3.09 (1.20-7.96)	0.020	0.19 (0.02-1.61)	0.303
Secondary	2.81 (1.52-5.18)	0.001	2.00 (0.53-7.51)	0.126
Tertiary	1.00			
**Mother’s working status**				
Housewife	1.64 (1.00-2.67)	0.049	0.61 (0.21-1.72)	0.347
Working	1.00			
**Household poverty status**				
Poor	3.15 (1.65-6.04)	0.001	1.18 (0.28-4.89)	0.822
Low income	2.27 (1.26-4.08)	0.006	1.82 (0.59-5.68)	0.301
Non-poor	1.00			
**Number of children**				
3 persons and below	1.00			
4 persons and above	2.02 (1.16-3.53)	0.013	5.86 (1.96-17.55)	0.002
**Child’s sex**				
Female	2.11 (1.30-3.43)	0.030	1.42 (0.62-3.27)	0.406
Male	1.00			
**Household food security status**				
Household food secure	1.00			
Household food insecure	0.93 (0.50-1.70)	0.802	0.78 (0.28-2.19)	0.632
Individual food insecure	2.10 (1.09-4.06)	0.027	1.08 (0.30-3.87)	0.908
Child hunger	19.92(4.55-87.32)	<0.001	16.38 (1.34-199.7)	0.028
**Child’s dietary intake:**			.	
Kilocalories (kcal)	0.993 (0.992-0.995)	<0.001	0.988 (0.98-0.99)	<0.001
Protein (g)	0.931 (0.91-0.95)	<0.001	1.06 (1.01-1.12)	0.035
Vitamin A (RE)	0.998 (0.997-0.999)	<0.001	0.999 (0.997-1.00)	0.045
Iron (mg)	0.83 (0.78-0.88)	<0.001	1.12 (0.98-1.28)	0.091
**Frequency of child illness (fever/diarrhoea/flu)**				
Every month	3.83 (2.09-7.00)	<0.001	2.79 (1.06-7.31)	0.037
Once in 2 months	1.67 (0.82-3.39)	0.158	0.60 (0.17-2.18)	0.438
Rarely (>3 months)	1.00			
**Child’s birth weight**				
Low birth weight	6.37 (2.72-14.94)	<0.001	6.83 (1.62-28.89)	0.009
**History of child’s worm infection (yes)**	2.27 (1.28-4.03)	0.005	3.48 (1.25-9.70)	0.017
**Mother’s personally own asset (yes)**	2.18 (1.33-3.57)	0.002	0.84 (0.30-2.40)	0.747
**BMI status of the mother**				
Normal weight	1.00			
Underweight	6.34 (1.37-29.34)	0.018	7.26 (0.65-80.78)	0.107
Overweight/obesity	0.73 (0.44-1.20)	0.216	0.48 (0.21-1.12)	0.089

Note. case = moderate to severe malnourished preschool children with z-scores < −2SD from the median of WHO reference based on visits to health clinics of Terengganu, Malaysia between April- August 2012; control = child without malnutrition with z-scores between -2SD and +2SD, age- matched and selected from the same health clinic as case; sd = standard deviation; CI = confidence interval; primary education and less = attained less than 6 years of school; secondary education = attained 11 years of education; tertiary education = obtained certificate, diploma, first degree and higher degree qualifications at academic and professional fields; poor = income per capita ≤ RM198; low income = RM198 < income per capita ≤ RM357; non-poor = income per capita > RM357; low birth weight = birth weight < 2.5 kg; BMI = body mass index; normal weight = BMI 18.5-22.9 kg/m^2^; underweight = BMI < 18.5 kg/m^2^; overweight/obesity = BMI ≥ 23 kg/m^2^; significance considered when p < 0.05 and if the OR and aOR estimate did not cross the null for 95% CI; AOR^1^ = Adjusted odds ratios for mother’s education level, mother’s working status, household poverty status, child’s sex, dietary iron intake, mother’s personally own asset and mother’s BMI.

## Discussion

In general, it was found that more malnourished children were female, had more siblings, and came from poor and food insecure families. A higher proportion of mothers of malnourished children, as compared to those with healthy children, were housewives, attained lower educational level, underweight, and did not own any personal assets. Malnourished children also suffered from frequent illness, with a history of worm infection and had low birth weight.

While food availability is not the main issue for Malaysia, household’s food accessibility remains a great challenge. The situation is aggravated by the recent food price hike [26]. Child hunger indicates the most severe form of food insecurity since adult caregivers are assumed to cut down their own food intake first in order to maintain an adequate food provision for their children [27]. These findings support the results from previous studies in Colombia [28] and Nigeria [29]. Zalilah and Ang [22] however, reported contradicting results in a study conducted among low-income households in Kuala Lumpur, Malaysia [22]. As reported by the authors, the contradicting results were due to underreporting of household food security status by the mothers because of embarrassment. Food insecurity might not necessarily result in poor nutritional status as there were presence of other risk factors such as diet inadequacy, recurrent infections, birth weight and socio-economic status that affected children’s health status directly but were not taken into account in the study.

In this study, a majority of the malnourished children suffered from multiple macronutrients and micronutrients deficiencies. This is consistent with other studies [30–32]. When compared with the recommended nutrient intake (RNI) of Malaysia, the dietary intakes of cases were inadequate in energy and vitamin A. Similar finding was reported among malnourished children in Kelantan, Malaysia [14]. It was found that many of the cases had low intake of staple foods especially rice, noodles and bread. The low calorie intake could be due to poor access to food, poor feeding practices and frequent illness. Furthermore, the significantly lower intake of micronutrients reflected poor diet quality among the malnourished children. The cases were found to consume less fruits, green-leafy vegetables, milk and dairy products, but consumed more unhealthy snacks and sweet drinks.

In addition, having more children was shown to be a significant predictor for childhood malnutrition even after adjustment for other confounders in the multivariate analyses. A similar finding was reported in studies in Malaysia [33], Pakistan [34] and Vietnam [35]. The increased number of children in families placed a heavy burden on the scarce household resources, particularly on financial and food; it also reduced the time and quality of care received by the children [36].

This study demonstrated that frequent illness and infections were important determinants for child malnutrition. These results are consistent with other studies in Laos, Nicaragua and Vietnam [37–39]. A previous study in Malaysia, however, did not find any associations between children stunting, wasting and underweight with children frequency of illness [14]. In addition, worm infection was also significantly associated with children’s nutritional status. During data collection, it was observed that some of the mothers allowed their children to walk or play barefooted at the playground. Okyay et al. suggested that low socio-economic status, poor sanitation and low maternal education were among the factors facilitating worm transmission among children [40]. In accordance with other studies [14, 37, 41, 42], low birth weight was found to be a risk factor of child malnutrition. Similarly, neonatal weight and length were found to be the strongest determinant of children’s weight and height status in a study conducted in the rural areas of Indonesia [43].

Similar with other studies in Malaysia [12–15], poverty was common among households with malnourished children. Lower income among cases was probably related to the occupation of their fathers and working status of their mothers. The common occupations among the fathers of malnourished children were labour workers in construction sites, fishermen, rubber tappers, technicians, drivers and village workers. A majority of the mothers of malnourished children were housewives who did not own any personal assets. Household poverty status, however, was not found to be a significant predictor of childhood malnutrition, which was similarly reported in other studies [35, 44]. It was found that a significant proportion of malnourished children came from non-poor families. Similar results were reported by several studies in Malaysia [12–14]. Possible explanations include differences in child caring practices and resources, household allocation of resources as well as low awareness about nutrition, hygiene and sanitation.

Despite the accumulative evidences on the importance of water and sanitation on children’s health [8, 30, 45, 46], our study did not find any association between these variables. A possible reason could be that Malaysia has reasonably good quality of water and sanitation, thus no association was observed. This is consistent with previous studies in some developing countries [35, 47–50].

This study did not find any significant associations between children’s nutritional status with breast feeding and the time complementary feeding was introduced. Similar findings were reported in India [51] and Malaysia [14]. This was probably due to the widespread breast feeding practices among the respondents who were Malays. Earlier study in Malaysia indicated that breast feeding practice among children less than one year old was widespread, especially among the rural Malay women with a prevalence of 96.7% [52]. In contrast, more mothers of controls were working which made exclusive breast feeding more difficult to practise.

There are some limitations in this study that warrant discussion. First, its observational design does not allow causality to be established. Second, data collection was confined to only health clinics in one state of Malaysia. Therefore, the study population may not represent the whole country. This study should be extended to different states of Malaysia and to be carried out in a prospective manner to enable better generalization of the findings.

## Conclusions

In conclusion, food insecurity at child level, low birth weight, frequent infection, inadequate nutrients intake, and large number of children were significant predictors of childhood malnutrition. Some variables which were found to be significant only at univariate analyses included income poverty, low maternal education, nonworking mothers, female children, underweight mothers, and lack of maternal ownership of asset. Therefore, it is recommended that emphasis should be focused on poverty reduction, improved access to health facilities, community-based nutrition and hygiene education programmes. Comprehensive interventions such as improving the nutritional status of pregnant mothers, improving quality of complementary feeding for children together with extensive family planning programmes and de-worming programmes are necessary to combat childhood malnutrition.

## Abbreviations

MCH: Maternal and child health
SD: Standard deviation
OR: Odds ratio
aOR: Adjusted odds ratio
CI: Confidence interval
BMI: Body mass index
PLI: Poverty line income
WHO: World Health Organization
RNI: Recommended nutrient intake
EI: Energy intake
BMR: Basal metabolic rate
WAZ: Weight for age z-score
HAZ: Height for age z-score
WHZ: Weight for height z-score.

## References

[1] UNICEF, WHO, World Bank: **Levels & trends in child malnutrition: Unicef-WHO-The World Bank Joint Estimates.** http://www.who.int/nutgrowthdb/jme_unicef_who_wb.pdf

[2] UNICEF: **Strategy for improved nutrition of children and women in developing countries.** http://www.google.com.my/url?sa=t&rct=j&q=&esrc=s&source=web&cd=1&ved=0CB0QFjAA&url=http%3A%2F%2Fwww.ceecis.org%2Fiodine%2F01_global%2F01_pl%2F01_01_other_1992_unicef.pdf&ei=aXvjU4KLNYyLuATUroJw&usg=AFQjCNFGIY6t8EixHWRT0R0tg2vG_J0opw&bvm=bv.72676100,d.c2E 10.1007/BF02810402 1937618

[3] UNICEF: **The state of the world’s children.** http://www.unicef.org/sowc98/

[4] Engle PL, Menon P, Haddad L (1999). Care and nutrition: concepts and measurement. World Devel.

[5] Pongou R, Ezzati M, Salomon J (2006). Household and community socioeconomic and environmental determinants of child nutritional status in Cameroon. BMC Public Health.

[6] Deolalikar AB (2005). Poverty and child malnutrition in Bangladesh. J Develop Soc.

[7] Thang NM, Popkin B (2003). Child malnutrition in Vietnam and its transition in an era of economic growth. J Hum Nutr Diet.

[8] Islam MM, Alam M, Tariquzaman M, Kabir MA, Pervin R, Begum M, Khan MM (2013). Predictors of the number of under-five malnourished children in Bangladesh: application of the generalized poisson regression model. BMC Public Health.

[9] Agee MD (2010). Reducing child malnutrition in Nigeria: combined effects of income growth and provision of information about mothers’ access to health care services. Soc Sci Med.

[10] Gross R, Schultink W, Sastroamidjojo S (1996). Stunting as an indicator for health and wealth: an Indonesian application. Nutr Res.

[11] Ruel MT, Levin CE, Armar-Klemesu M, Maxwell D, Morris SS (1999). Good care practices can mitigate the negative effects of poverty and low maternal schooling on children’s nutritional status: evidence from Accra. World Devel.

[12] Chee HL, Khor GL, Fatimah A, Wan Manan WM, Mohd Nasir MT, Nik Shanita S, Norimah AK, Norlela MH, Normah H, Poh BK, Rokiah MY (2002). Nutritional assessment of pre-school children in rural villages of the family dynamics, lifestyles and nutrition study (1997–2001) II prevalence of undernutrition and relationship to household socio-economic indicators. Malays J Nutr.

[13] Zamaliah MM, Khor GL, Tee ES (2002). Socio-economic determinants of nutritional status of children in rural Peninsular Malaysia. Asia Pac J Clin Nutr.

[14] Cheah WL, Wan Manan WM, Zabidi Azhar MH, Chang CT (2010). Factors associated with undernutrition among children in a rural district of Kelantan, Malaysia. Asia Pac J Public Health.

[15] Norhayati M, Noorhayati MI, Mohammod CG, Oothuman P, Azizi O, Fatimah A, Fatmah MS (1997). Malnutrition and its risk factors among children 1–7 years old in rural Malaysian communities. Asia Pac J Clin Nutr.

[16] United Nations Development Group (2010). Thematic papers on the millenium development goals.

[17] Malaysia Health Informatics Centre (2012). Data on Children Receiving Food Basket Between According to States Between 2009 and 2011.

[18] Malaysia Department of Statistics (2010). Population distribution and basic demographic characteristics.

[19] de Onis M, Blössner M, World Health Organization: **WHO global database on child growth and malnutrition.** libdoc.who.int/hq/1997/WHO_NUT_97.4.pdf 10.1093/ije/dyg099 12913022

[20] Cheah WL, Wan Abdul Manan W, Zabidi Azhar MH, Chang CT (2009). Development of a questionnaire for the study of malnutrition among children in rural Kelantan. Malaysia Rural Remote Health.

[21] Malaysia Department of Statistics (2009). Household income and basic amenities survey report.

[22] Zalilah M, Ang M (2001). Assessment of food insecurity among low income households in Kuala Lumpur using the Radimer/Cornell food insecurity instrument—a validation study. Malays J Nutr.

[23] Goldberg GR, Black AE (1998). Assessment of the validity of reported energy intakes-review and recent developments. Food Nutr Res.

[24] Livingstone MBE, Black AE (2003). Markers of the validity of reported energy intake. J Nutr.

[25] **WHO Anthro** http://www.who.int/childgrowth/software/en/

[26] Malaysia Department of Statistics (2013). Consumer price index Malaysia November.

[27] Radimer KL, Olson CM, Greene JC, Campbell CC, Habicht JP (1992). Understanding hunger and developing indicators to assess it in women and children. J Nutr Educ.

[28] Hackett M, Melgar-Quinonez H, Alvarez MC (2009). Household food insecurity associated with stunting and underweight among preschool children in Antioquia, Colombia. Pan Am J Public Health.

[29] Ajao KO, Ojofeitimi EO, Adebayo AA, Fatusi AO, Afolabi OT (2010). Influence of family size, household food security status, and child care practices on the nutritional status of under-five children in Ile-Ife, Nigeria. Afr J Reprod Health.

[30] Ahmed AM, Ahmed T, Roy SK, Alam N, Hossain MI (2012). Determinants of undernutrition in children under 2 years of age from rural Bangladesh. Indian Pediatr.

[31] Donnen P, Brasseur D, Dramaix M, Vertongen F, Ngoy B, Zihindula M, Hennart P (1996). Vitamin A deficiency and protein-energy malnutrition in a sample of pre-school age children in the Kivu Province in Zaire. Eur J Clin Nutr.

[32] Khor GL (2003). Update on the prevalence of malnutrition among children in Asia. Nepal Med Coll J.

[33] Soon SD, Khor GL (1995). Nutritional status of children aged one to six years in Sg. Koyan FELDA in Pahang. Malays J Nutr.

[34] Khattak M, Ali S (2010). Malnutrition and associated risk factors in pre-school children (2–5 Years) in District Swabi (NWFP)-Pakistan. J Med Sci.

[35] Hien NN, Kam S (2008). Nutritional status and the characteristics related to malnutrition in children under five years of age in Nghean, Vietnam. J Prev Med Public Health.

[36] Senbanjo IO, Olayiwola IO, Afolabi WA, Senbanjo OC (2013). Maternal and child under-nutrition in rural and urban communities of Lagos state, Nigeria: the relationship and risk factors. BMC Res Notes.

[37] Jesmin A, Yamamoto SS, Malik AA, Haque MA (2011). Prevalence and determinants of chronic malnutrition among preschool children: a cross-sectional study in Dhaka City, Bangladesh. J Health Popul Nutr.

[38] Kamiya Y (2011). Socioeconomic determinants of nutritional status of children in Lao PDR: effects of household and community factors. J Health Popul Nutr.

[39] Sakisaka K, Wakai S, Kuroiwa C, Flores LC, Kai I, Mercedes Arago’n M, Hanada K (2006). Nutritional status and associated factors in children aged 0–23 months in Granada, Nicaragua. Public Health.

[40] Okyay P, Ertug S, Gultekin B, Onen O, Beser E (2004). Intestinal parasites prevalence and related factors in school children, a western city sample-Turkey. BMC Public Health.

[41] Alasfoor D, Traissac P, Gartner A, Delpeuch F (2007). Determinants of persistent underweight among children, aged 6–35 months, after huge economic development and improvements in health services in Oman. J Health Popul Nutr.

[42] Tharakan CT, Suchindran CM (1999). Determinants of child malnutrition—an intervention model for Botswana. Nutr Res.

[43] Schmidt MK, Muslimatun S, West CE, Schultink W, Gross R, Hautvast JG (2002). Nutritional status and linear growth of Indonesian infants in west java are determined more by prenatal environment than by postnatal factors. J Nutr.

[44] Larrea C, Kawachi I (2005). Does economic inequality affect child malnutrition? The case of Ecuador. Soc Sci Med.

[45] Psaki S, Bhutta ZA, Ahmed T, Ahmed S, Bessong P, Islam M, John S, Kosek M, Lima A, Nesamvuni C, Shrestha P, Svensen E, McGrath M, Richard S, Seidman J, Caulfield L, Miller M, Checkley W (2012). Household food access and child malnutrition: results from the eight-country MAL-ED study. Popul Health Metr.

[46] Sharghi A, Kamran A, Faridan M (2011). Evaluating risk factors for protein-energy malnutrition in children under the age of six years: a case–control study from Iran. Int J Gen Med.

[47] Beiersmann C, Bermejo Lorenzo J, Bountogo M, Tiendrebeogo J, Gabrysch S, Ye M, Jahn A, Muller O (2013). Malnutrition determinants in young children from Burkina Faso. J Trop Pediatr.

[48] Kabubo-Mariara J, Ndenge GK, Mwabu DK (2009). Determinants of children’s nutritional status in Kenya: evidence from demographic and health surveys. J Afr Economies.

[49] Masiye F, Chama C, Chitah B, Jonsson D (2010). Determinants of child nutritional status in Zambia: an analysis of a national survey. Zambia Soc Sci J.

[50] Van de Poel E, Hosseinpoor AR, Jehu-Appiah C, Vega J, Speybroeck N (2007). Malnutrition and the disproportional burden on the poor: the case of Ghana. Int J Equity Health.

[51] Menon P, Bamezai A, Subandoro A, Ayoya MA, Aguayo V (2013). Age-appropriate infant and young child feeding practices are associated with child nutrition in India: insights from nationally representative data [abstract]. Matern Child Nutr.

[52] Fatimah S, Siti Saadiah HN, Tahir A, Hussain Imam MI, Ahmad Faudzi Y (2010). Breastfeeding in Malaysia: results of the third national health and morbidity survey (NHMS III) 2006. Malays J Nutr.

[CR53] The pre-publication history for this paper can be accessed here:http://www.biomedcentral.com/1471-2458/14/785/prepub

